# Rapid, high-sensitivity detection of biomolecules using dual-comb biosensing

**DOI:** 10.1038/s41598-023-41436-3

**Published:** 2023-09-26

**Authors:** Shogo Miyamura, Ryo Oe, Takuya Nakahara, Hidenori Koresawa, Shota Okada, Shuji Taue, Yu Tokizane, Takeo Minamikawa, Taka-Aki Yano, Kunihiro Otsuka, Ayuko Sakane, Takuya Sasaki, Koji Yasutomo, Taira Kajisa, Takeshi Yasui

**Affiliations:** 1 Graduate School of Advanced Technology and Science, Tokushima University, 2-1 Minami-Josanjima, Tokushima, Tokushima 770-8506 Japan; 2 Graduate School of Sciences and Technology for Innovation, Tokushima University, 2-1 Minami-Josanjima, Tokushima, Tokushima 770-8506 Japan; 3 School of System Engineering, Kochi University of Technology, 185 Miyanokuchi, Tosayamada, Kami, Kochi 782-8502 Japan; 4 Division of Next-Generation Photonics, Institute of Post-LED Photonics (pLED), Tokushima University, 2-1 Minami-Josanjima, Tokushima, Tokushima 770-8506 Japan; 5 Division of Interdisciplinary Researches for Medicine and Photonics, Institute of Post-LED Photonics (pLED), Tokushima University, 2-1 Minami-Josanjima, Tokushima, Tokushima 770-8506 Japan; 6 Department of Immunology and Parasitology, Graduate School of Medicine, Tokushima University, 3-18-15 Kuramoto, Tokushima, Tokushima 770-8503 Japan; 7 Department of Biochemistry, Graduate School of Medicine, Tokushima University, 3-18-15 Kuramoto, Tokushima, Tokushima 770-8503 Japan; 8 Graduate School of Interdisciplinary New Science, Toyo University, 2100 Kujirai, Kawagoe, Saitama 350-8585 Japan

**Keywords:** Optical sensors, Imaging and sensing, Biophotonics, Frequency combs

## Abstract

Rapid, sensitive detection of biomolecules is important for biosensing of infectious pathogens as well as biomarkers and pollutants. For example, biosensing of severe acute respiratory syndrome coronavirus 2 (SARS-CoV-2) is still strongly required for the fight against coronavirus disease 2019 (COVID-19) pandemic. Here, we aim to achieve the rapid and sensitive detection of SARS-CoV-2 nucleocapsid protein antigen by enhancing the performance of optical biosensing based on optical frequency combs (OFC). The virus-concentration-dependent optical spectrum shift produced by antigen–antibody interactions is transformed into a photonic radio-frequency (RF) shift by a frequency conversion between the optical and RF regions in the OFC, facilitating rapid and sensitive detection with well-established electrical frequency measurements. Furthermore, active-dummy temperature-drift compensation with a dual-comb configuration enables the very small change in the virus-concentration-dependent signal to be extracted from the large, variable background signal caused by temperature disturbance. The achieved performance of dual-comb biosensing will greatly enhance the applicability of biosensors to viruses, biomarkers, environmental hormones, and so on.

## Introduction

Biosensors are biomolecular sensors that utilize or imitate the skillful molecular identification function of living organisms; they are applied to a wide range of fields such as medical care, food industry, and environmental monitoring. Yet, further enhancement of biosensing performance is still required in the field of infectious pathogens as well as biomarkers, pollutant, bacteria, and environmental hormones. For example, one timely and urgent application that benefits from improved performance is still testing of coronavirus disease 2019 (COVID-19) because COVID‐19, caused by severe acute respiratory syndrome coronavirus 2 (SARS-CoV-2), has rapidly spread and is still occurring all over the world. While reverse-transcription polymerase chain reaction (RT-PCR)^1–3^ has been widely used as a current standard for COVID-19 testing in clinical practice, research has also been conducted on biosensors of SARS-CoV-2, vigorously. The potential methods that may improve the biosensing performance is the use of optical biosensors^4,5^ due to both rapidity and high sensitivity. For example, optical biosensors based on surface plasmon resonance (SPR)^6,7^ have been widely used for analyzing biomolecules and viruses; in this technique, the spectral shift of the SPR trough in the wavelength or angular spectrum is measured because the spectral shift depends on a sample concentration due to a combined effect of SPR and molecular identification function on the sensor surface. SPR analysis enables the real-time, label-free analysis of intermolecular interaction or combination by measuring a sample-concentration-dependent optical spectral shift; thus, this technique has been widely applied for the detection of various infectious pathogens such as human immunodeficiency virus ^8^, Ebola virus^9^, norovirus^10^, influenza virus^11^, and even SARS-CoV-2^12–14^ as well as various biomarkers such as proteins^15,16^, DNA^17,18^, and whole cells^19,20^. The limit of detection (LOD) for SARS-CoV-2 nucleocapsid protein (N protein) antigen has reached 85 fM or 4 pg/ml^14^; however, in order to measure clinical samples (for example, human nasopharyngeal aspirates), further improvement in LOD (to the fM level or sub-fM level) is necessary^21^. A reason for the limited sensitivity in SPR is an optical instrumentation resolution as well as a relatively broad spectrum of SPR trough compared to its slight spectrum shift. Optical biosensing techniques, such as fluorescence^22,23^ and surface-enhanced Raman scattering (SERS)^24,25^, could be another potential candidate for the biosensing of SARS-CoV-2. For instance, a combination of SERS with antibody pair, SERS-active hollow Au nanoparticles, and magnetic beads achieved LOD of 2.56 fg/mL for the SARS-CoV-2 antigen, facilitating the identification of SARS-CoV-2 in human nasopharyngeal aspirates within 30 min^21^. While the discrimination of infection has been achieved, the Raman signals of samples diagnosed as negative still show variations among samples, which may lead to potential misdiagnosis between false positive and true negative during the early stages of infection.

Regarding the limitation of optical instrumentation resolution in SPR, if this sample-concentration-dependent optical spectral shift is transformed into a photonic radio-frequency (RF) signal, then such photonic RF biosensing would benefit from the high precision and real-time nature provided by well-established electric frequency measurements with RF frequency standards and measurement apparatuses. Recently, optical frequency combs (OFCs)^26–29^ have attracted attention for use as photonic RF sensors based on a frequency conversion function between the optical and RF regions^30,31^. OFC is composed of a series of optical frequency modes (freq. = *ν*_*m*_) with a constant mode spacing of *f*_*rep*_ in the RF band. A relation between *ν*_*m*_ and *f*_*rep*_ is given by

1 $$\nu_{m} = f_{ceo} + mf_{rep,}$$

where *f*_*ceo*_ is a carrier-envelope-offset frequency and *m* is mode number. Since *f*_*rep*_ is a RF signal, OFC acts as an accurate frequency converter between optical and electrical regions. For example, a refractive-index-dependent (RI-dependent) optical spectrum shift was converted into a change in *f*_*rep*_ of around several tens of MHz by placing a multimode-interference (MMI) fiber sensor^32,33^ inside a fiber OFC cavity^34–36^. Then, the *f*_*rep*_ signal with the spectral linewidth below 1 Hz was rapidly and precisely measured by a RF frequency counter. Furthermore, the intracavity fiber sensor enables multiple interactions between the sample and the light, enhancing the sensitivity. Due to the precise electric-measurement of the narrow-linewidth, sensitivity-enhanced *f*_*rep*_ signal, this RI-sensing OFC enables precise measurement of the sample RI with a resolution of 4.88 × 10^−6^ refractive index units (RIU)^34^, which is two orders of magnitude better than that of the previous study of RI sensing with MMI fiber sensor^32^. Such high-sensitivity RI-sensing OFCs would have the potential to be further extended to optical biosensing, namely, to biosensing OFCs, through surface modification of the MMI fiber sensor with the molecular identification layer in terms of biomolecule interactions, similar to the surface modification employed in SPR. However, there are no attempts to apply this RI-sensing OFC for optical biosensing because the residual temperature drift of the *f*_*rep*_ signal (typically, a few hundreds Hz/hour) is still larger than the sample-concentration-dependent *f*_*rep*_ shift in biosensing (typically, a few to a few tens Hz), reducing the precision and hindering its extension to the biosensing OFC. It is essential to largely reduce the temperature drift of *f*_*rep*_ in order to open the door for the biosensing OFC.

Thus, in this article, we first developed a dual-comb configuration with an active sensing OFC and a dummy sensing OFC to suppress the temperature drift of the *f*_*rep*_ signal, namely, dual-comb biosensing; this function is similar to the active-dummy temperature compensation of strain sensors. Then, for preliminarily test of dual-comb biosensing, we applied the active-dummy dual sensing OFCs to RI sensing of a glycerol solution, which is a stable standard substance without volatilization. Finally, for proof of concept using biomolecules, we demonstrate rapid detection of the SARS-CoV-2 N protein antigen by combining the active-dummy dual sensing OFCs and surface modification of a SARS-CoV-2 N protein antibody. Prior to using real samples of SARS-CoV-2, to assess the net performance of dual-comb biosensing alone without the help of specific enhancing techniques regarding antigen–antibody interactions and/or surface modifications, commercially available, purified SARS-CoV-2 N protein antigen diluted in phosphate-buffered saline (PBS) was detected by a help of a commercially available SARS-CoV-2 N protein antibody. Since the dual-comb biosensing can be implemented with a wide variety of surface modifications for other viruses/pathogens and biological molecules, it greatly enhances the applicability of optical biosensors for virus, bacteria, protein, biomarker, environmental hormone, and so on.

## Results

### General principle of operation

**Figure 1:**
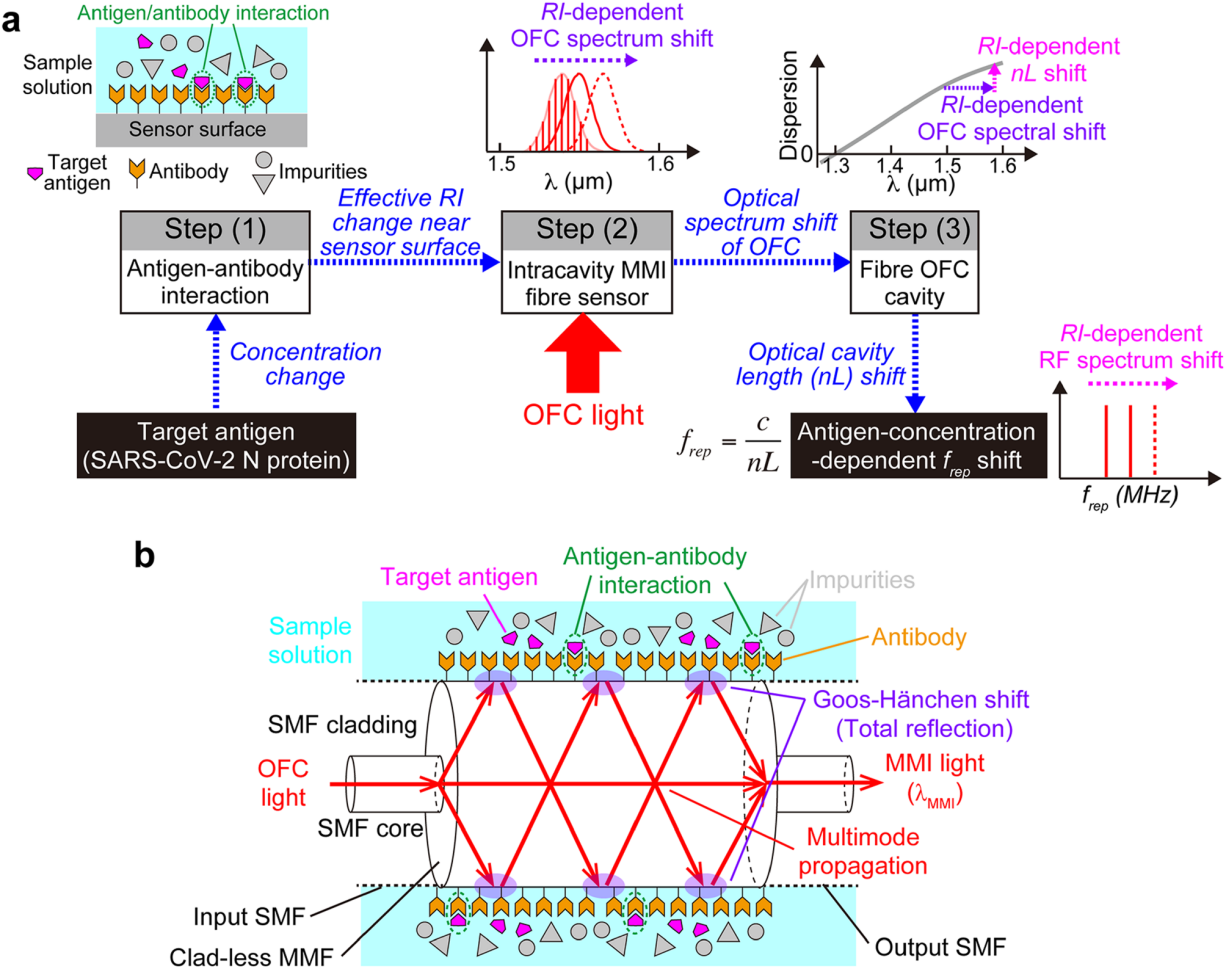
Principle of operation for the biosensing OFC. (**a**) Block diagram of the signal flow. The concentration of the target antigen is obtained according to the mode spacing *f*_*rep*_ of the OFC through three steps: Step (1) antigen–antibody interactions on the antibody-modified sensor surface, Step (2) RI-dependent optical spectrum shift of OFC provided by the intracavity MMI fiber sensor, and Step (3) photonic-to-RF conversion by the wavelength dispersion of the fiber cavity. (**b**) Schematic diagram of the intracavity MMI fiber sensor with antibody surface modification. The antigen–antibody interaction on the surface of the intracavity MMI fiber sensor is reflected by an effective RI change near the sensor surface, while the intracavity MMI fiber sensor functions as an RI-dependent variable-optical-bandpass filter via the MMI process. The combination of the antigen–antibody interaction with the MMI fiber sensor causes an antigen-concentration-dependent optical spectrum shift of OFC.

In this study, we sought to design a biosensor that combines photonic-to-RF conversion and antigen–antibody interaction in an OFC. The biosensing OFC operates through three steps: Step (1) antigen–antibody interactions on the antibody-modified sensor surface, Step (2) RI-dependent optical spectrum shift of OFC provided by the intracavity MMI fiber sensor, and Step (3) photonic-to-RF conversion by the wavelength dispersion of the fiber cavity, as depicted in Fig. 1a. In Step (1), the selective combination of a target antigen with the corresponding antibody changes the effective RI near the sensor surface depending on the antigen concentration. In Step (2), since the intracavity MMI fiber sensor transmits only certain wavelength (*λ*_*MMI*_) light based on its RI due to MMI and the Goos-Hänchen shift, the OFC shows an RI-dependent and hence an antigen-concentration-dependent shift in the optical spectrum. Simultaneously, the intracavity MMI fiber sensor enhances the sensing sensitivity by multiple interactions between light and sample inside the OFC cavity. The functions of Steps (1) and (2) are implemented by an intracavity MMI fiber sensor with antibody surface modification, as shown in Fig. 1b. In Step (3), the antigen-concentration-dependent shift in the optical spectrum is converted to a shift in the optical cavity length *nL*, where *n* and *L* are the RI and the physical length of the OFC cavity fiber, via the wavelength dispersion of RI in the cavity fiber. Finally, the change in the antigen concentration can be read out as the *f*_*rep*_ shift via *f*_*rep*_ = *c/nL*, where *c* is the velocity of light in a vacuum. Importantly, the *f*_*rep*_ linewidth achieves down to below 1 Hz, which is smaller than *f*_*rep*_ shift expected due to the antigen concentration change.

### Temperature drift in the single-comb configuration

**Figure 2:**
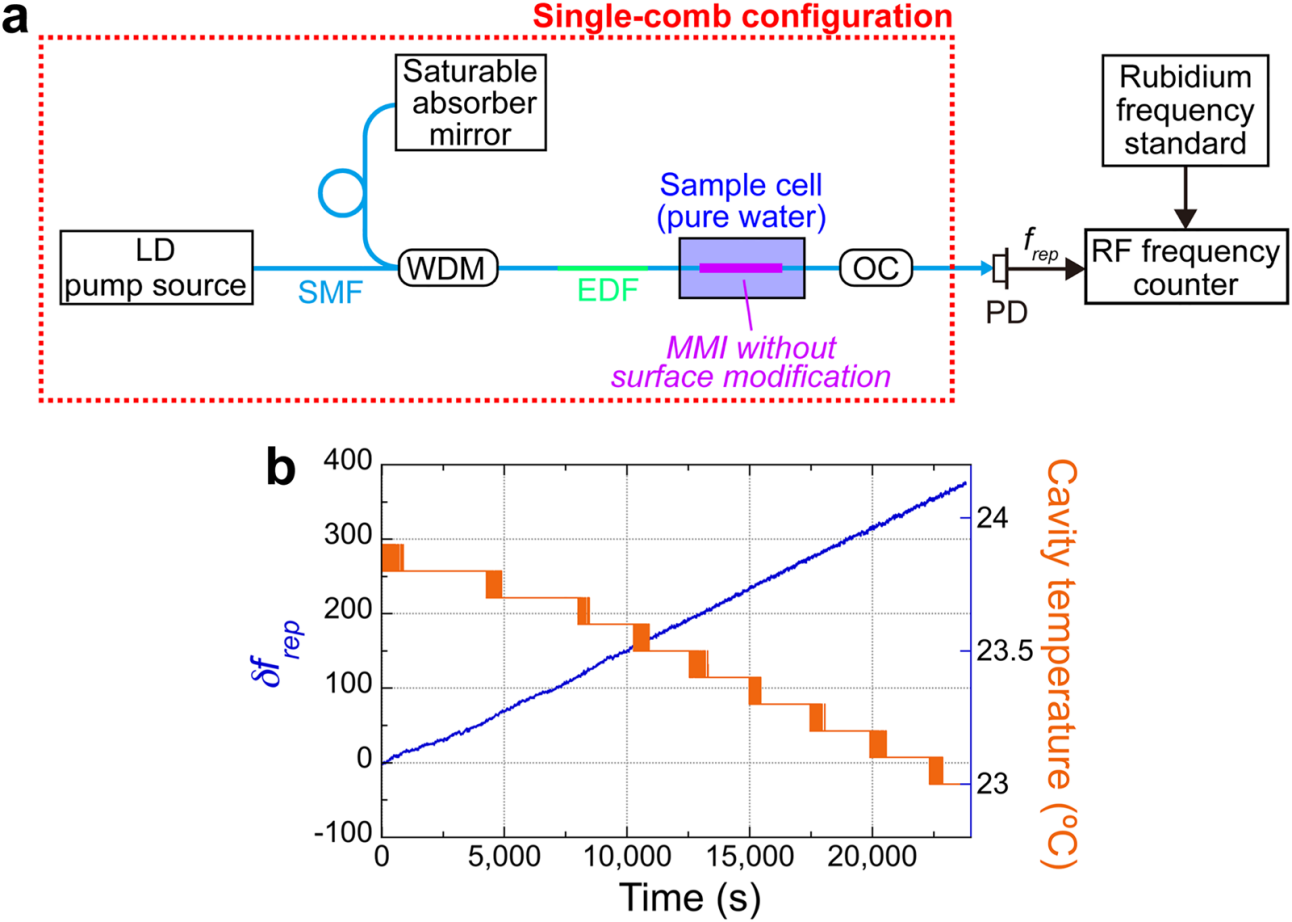
Basic performance of single-comb RI sensing of pure water with temperature drift. (**a**) Schematic drawing of the experimental setup. *LD* laser diode; *SMF* single-mode fiber; *WDM* wavelength-division-multiplexing coupler; *EDF* erbium-doped fiber; *MMI* multimode-interference fiber sensor; *OC* fiber output coupler; *PD* photodiode. Details of the single sensing OFC are given in the Materials and Methods section. (**b**) Temporal drift of the cavity temperature (orange line) and the corresponding *f*_*rep*_ shift (*δf*_*rep*_, blue line). Pure water was used as the sample of RI sensing. *δf*_*rep*_ was calculated as the frequency deviation from the initial value of *f*_*rep*_. The stepped-down behavior of the cavity temperature is due to the temperature resolution of the thermistor (= 0.1 °C) used for monitoring the cavity temperature. The cavity temperature sensitivity of the single sensing OFC was approximately − 400 Hz/°C.

We first evaluated the dependence of *f*_*rep*_ in a single sensing OFC on cavity temperature because the temperature disturbance to the fiber OFC cavity fluctuates *f*_*rep*_ via thermal expansion or shrinkage of *nL*. To this end, we measured the temporal drift in the *f*_*rep*_ of the single-comb configuration of sensing OFC under an uncontrolled cavity temperature, as shown in Fig. 2a, whose details are given in the Materials and Methods section. We set the center optical wavelength *λ*_*MMI*_ of 1556.6 nm and the frequency spacing *f*_*rep*_ of 31.7 MHz for stable mode-locked oscillation with the intracavity MMI fiber sensor. Pure water was used for a standard sample with a stable RI, and placed in a glass sample cell together with the MMI fiber sensor without the surface modification for RI sensing. The output light from the OFC was detected by a photodetector (PD), and *f*_*rep*_ was measured by an RF frequency counter synchronized to a rubidium frequency standard working in the RF band. Figure 2b shows the *f*_*rep*_ shift (*δf*_*rep*_, blue line) when the cavity temperature (orange line) changed over a range of 1 °C. *δf*_*rep*_ represents the frequency deviation from the initial measurement value. The temporal behavior of *δf*_*rep*_ in synchronization with the cavity temperature indicated a temperature sensitivity of approximately − 400 Hz/°C. Thus, the cavity-temperature-dependent *f*_*rep*_ drift is considerably larger than the sample-concentration-dependent *f*_*rep*_ shift in biosensing (typically, a few to a few tens Hz). Although the cavity temperature could be actively controlled within a range of 0.1 °C^34,36^, it is still insufficient to suppress the cavity-temperature-dependent *f*_*rep*_ drift (= 400 Hz/°C × 0.1 °C = 40 Hz) below the sample-concentration-dependent *f*_*rep*_ shift. Thus, to further reduce the temperature drift, we applied a dual-comb configuration for active-dummy compensation of the temperature drift as described in the following subsection.

### Active-dummy compensation of the temperature drift with the dual-comb configuration

**Figure 3:**
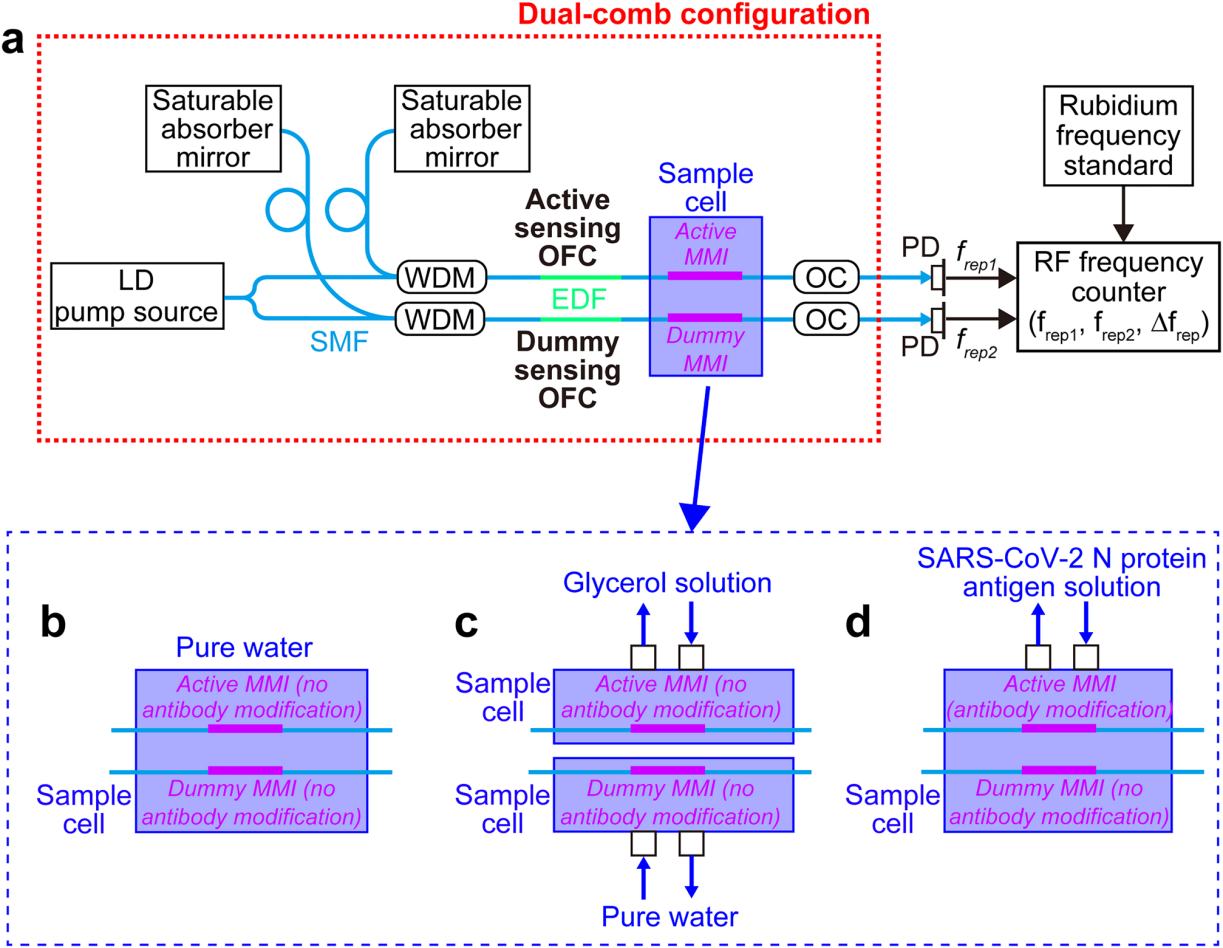
Experimental setup of dual-comb RI sensing and biosensing. (**a**) Schematic drawing of the whole experimental setup. *LD* laser diode; *SMFs* single-mode fibers; *OFCs* optical frequency combs; *WDMs* wavelength-division-multiplexing couplers; *EDF* erbium-doped fiber; *active MMI* intracavity multimode-interference fiber sensor for a target sample; *dummy MMI* intracavity multimode-interference fiber sensor for a reference sample; *OCs* fiber output couplers; *PDs* photodiodes. The active sensing OFC and dummy OFC operate at a center optical wavelength (*λ*_*MMI*_) of 1556.6 nm. (**b**) Schematic drawing of the MMI sensor and sample cell for dual-comb RI sensing of pure water. (**c**) Schematic drawing of the MMI sensor and sample cell for dual-comb RI sensing of glycerol solution. (**d**) Schematic drawing of the MMI sensor and sample cell for dual-comb biosensing of the SARS-CoV-2 N protein antigen solution. Peristaltic pumps were used for sample exchange in the dual-comb RI sensing of glycerol solution and the dual-comb biosensing of the SARS-CoV-2 N protein antigen.

A dual-comb configuration with an active sensing OFC with a frequency spacing of *f*_*rep1*_ and a dummy sensing OFC with a frequency spacing of *f*_*rep2*_ was adopted to compensate for the temperature drift. Figure 3a shows a schematic drawing of the dual-comb configuration, in which a pair of fiber OFC cavities were arranged in an aluminum box covered by insulated materials so that they were affected by similar temperature drifts ^37^. In this configuration, although *f*_*rep1*_ and *f*_*rep2*_ fluctuate depending on the residual drift of cavity temperature via thermal expansion or shrinkage of *nL*, their drifts are similar because they experience the same thermal disturbances. Therefore, the frequency difference *∆f*_*rep*_ between *f*_*rep1*_ and *f*_*rep2*_ remains constant regardless of the temperature drift of *f*_*rep1*_ and *f*_*rep2*_. Thus, when the active sensing OFC evaluates a sample solution in a certain temperature environment and the dummy sensing OFC evaluates a reference material in the same temperature environment, *∆f*_*rep*_ reflects the sample concentration without influence from temperature drift. In other words, a one-to-one correspondence between *∆f*_*rep*_ and the sample concentration is established independent of temperature drift. Figure 3b–d show the MMI and sample cell for dual-comb RI sensing of pure water, dual-comb RI sensing of glycerol solution, and dual-comb biosensing of the SARS-CoV-2 N protein antigen, respectively. Table 1 summarizes *λ*_*MMI*_, *f*_*rep1*_, *f*_*rep2*_, *∆f*_*rep*_, the MMI, and the sample cell used in the following three dual-comb sensing experiments; these values were selected for stable operation and better temperature compensation. A pair of output lights from the active and the dummy sensing OFCs is detected by a pair of photodetectors (PDs). Their frequency signals (= *f*_*rep1*_ and *f*_*rep2*_) and a frequency difference between them (= *∆f*_*rep*_ = *f*_*rep1*_ − *f*_*rep2*_) are measured by the RF frequency counter. Details of the dual-comb biosensing technique are given in the Materials and Methods section, together with details on the experimental and analytical methodology employed for all measurements.

**Table 1:** Experimental settings of dual-comb RI sensing and biosensing.

Experiments	*λ*_*MMI*_ (nm)	*f*_*rep1*_ (MHz)	*f*_*rep2*_ (MHz)	*∆f*_*rep*_ (kHz)	Active MMI	Dummy MMI	Sample cell
Dual-comb RI sensing of pure water	1556.6	31.7	32.5	− 851.9	No surface modification		Single [Fig. 3b]
Dual-comb RI sensing of glycerol solution	1556.6	31.7	32.5	− 851.9	No surface modification		Dual [Fig. 3c]
Dual-comb biosensing of SARS-CoV-2 N protein antigen	1556.6	29.6	29.7	− 88.6	Surface modification of antibody	No surface modification of antibody	Single [Fig. 3d]

**Figure 4:**
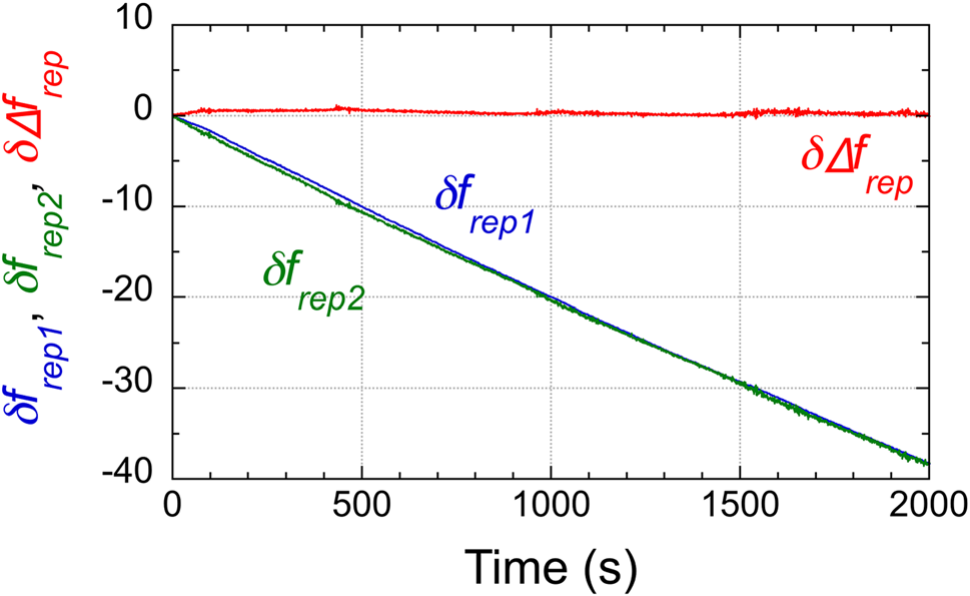
Basic performance of dual-comb RI sensing of pure water with temperature drift. Temporal drifts in *δf*_*rep1*_, *δf*_*rep2*_, and *δ∆f*_*rep*_ when pure water was used as a sample for the active and dummy RI-sensing OFCs [see the second row in Table 1 and Fig. 3b]. *δf*_*rep1*_, *δf*_*rep2*_, and *δ∆f*_*rep*_ were calculated as the frequency deviations from the initial values of *f*_*rep1*_, *f*_*rep2*_, and *∆f*_*rep*_, respectively.

The blue and green lines in Fig. 4 show the temporal shifts in *f*_*rep1*_ and *f*_*rep2*_, namely, *δf*_*rep1*_ and *δf*_*rep2*_, respectively, when pure water was used as a sample for both the active and dummy sensing OFCs without surface modification [see the second row in Table 1 and Fig. 3b]. *δf*_*rep1*_ and *δf*_*rep2*_ suffered from a frequency drift of over − 38 Hz, implying a temperature drift of 0.1 °C during the data acquisition from a temperature sensitivity of − 400 Hz/°C in Fig. 2b. However, importantly, *δf*_*rep1*_ and *δf*_*rep2*_ behaved almost the same in terms of drift. The resulting *∆f*_*rep*_ shift (*δ∆f*_*rep*_) was stable as shown by the red line in Fig. 4, in which the standard deviation of *δ∆f*_*rep*_ was 0.066 Hz at 10 s, 0.209 Hz at 100 s, and 0.177 Hz at 1000 s, respectively. These variations are equivalent to a temperature stability of 1.64 × 10^−4^ °C at 10 s, 5.23 × 10^−4^ °C at 100 s, and 4.42 × 10^−4^ °C at 1000 s, much better than that by the temperature controller of fiber OFC cavity.

**Figure 5:**
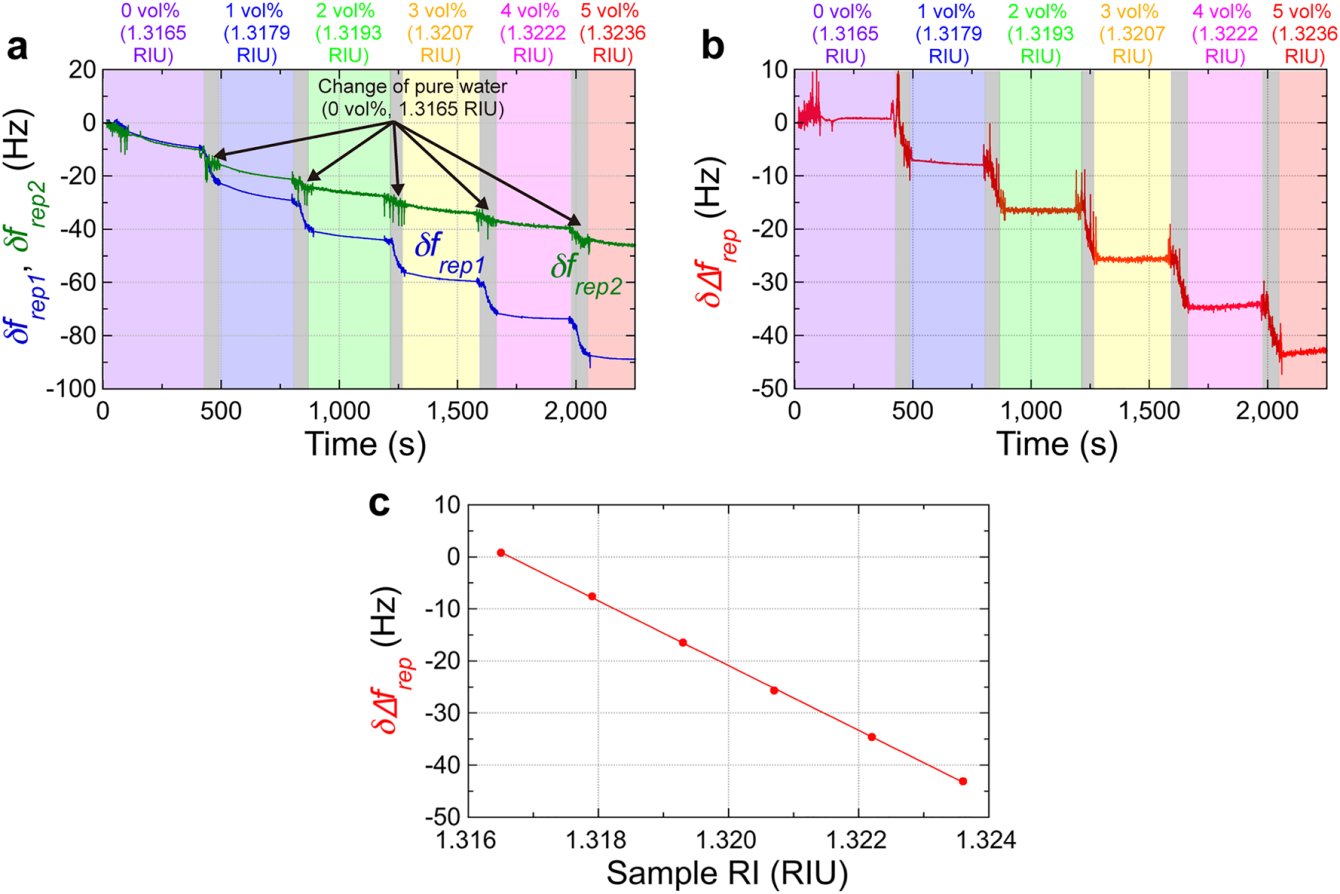
Temperature-drift-free dual-comb RI sensing of glycerol solution. (**a**) Sensorgrams of *δf*_*rep1*_ in the active sensing OFC and *δf*_*rep2*_ in the dummy sensing OFC. Glycerol solutions consisting of glycerin and pure water at different ratios were used as the target samples in the active sensing OFC; pure water was used as the reference sample in the dummy sensing OFC [see the third row in Table 1 and Fig. 3c]. Grey zones indicate the time period for sample exchange by peristaltic pumps.* δf*_*rep1*_ and *δf*_*rep2*_ were calculated as the frequency deviations from the initial values of *f*_*rep1*_ and *f*_*rep2*_, respectively. (**b**) Sensorgram of *δ∆f*_*rep*_ with mixtures of glycerin and pure water at different ratios. *δ∆f*_*rep*_ was calculated as the frequency deviation from the initial value of *∆f*_*rep*_. (**c**) Relationship between the sample RI and *δ*∆*f*_*rep*_.

We next tested active-dummy temperature compensation for RI sensing of a liquid sample different from the reference sample. For RI sensing, the active and dummy sensing OFCs have no surface modification in MMI [see the third row in Table 1 and Fig. 3c]. We used glycerol solutions consisting of glycerin and pure water at different ratios, corresponding to different RIs, as target samples in the active sensing OFC. This sample is easy to prepare and stable as it does not volatilize. We prepared six samples with different RIs [= 0 vol%, 1 vol%, 2 vol%, 3 vol%, 4 vol%, and 5 vol%, corresponding to 1.3165 RIU, 1.3179 RIU, 1.3193 RIU, 1.3207 RIU, 1.3222 RIU, and 1.3236 RIU; see color-highlighted zones in Fig. 5a,b] because their expected *f*_*rep*_ shift is comparable to the concentration-dependent *f*_*rep*_ shift caused by the SARS-CoV-2 N protein antigen. The RI of the sample was calculated from the volume ratio of water (RI = 1.3165 RIU at 1550 nm) and glycerin (RI = 1.4571 RIU at 1550 nm)^38^. While our experiments were conducted in volume ratio (vol%) of glycerin-water solutions, the correlation in the literature were provided in terms of weight ratio (wt%). To address this discrepancy, we performed a conversion between vol% and wt% by using the volume and density (= 1.26)^39^ of 100% glycerin to calculate the corresponding weight, and then dividing it by the weight of water. This process yielded the weight ratio. Subsequently, we employed this weight ratio to calculate the RI value of each sample based on the RI values from the literature. We exchanged the sample by using a peristaltic pump [see grey zones in Fig. 5a,b]. Additionally, pure water (a 0 vol% glycerol solution, corresponding to 1.3165 RIU) was used as a reference sample in the dummy sensing OFC. To prevent the temperature of the pure water in the dummy sample cell from increasing during repeated measurements, the pure water sample was exchanged with a new pure water sample with another peristaltic pump when the target sample was exchanged with a new RI glycerol sample. The blue and green lines in Fig. 5a represent *δf*_*rep1*_ and *δf*_*rep2*_ as the concentration of the glycerol solution increased from 0 vol% to 5 vol%. *δf*_*rep2*_, in the dummy sensing OFC, exhibited a slow drift with some rapid changes even though the RI of the pure water was constant. This slow drift is due to the temperature drift, too. Since rapid changes in *δf*_*rep2*_ were synchronized with the operation of the peristaltic pump, they are caused by disturbance from the water flow when the samples were exchanged. In contrast, *δf*_*rep1*_, in the active sensing OFC, exhibited a combination of a step-like change with the sample RI and the slow drift shown by *δf*_*rep2*_ together with rapid changes. This combination of behavior in *δf*_*rep1*_ is detrimental to the RI sensing performance in the single sensing OFC configuration. The behavior of rapid changes caused by the peristaltic pump is different between *δf*_*rep1*_ and *δf*_*rep2*_ because the shape of the two separate sample cells is not identical, and the position of the MMI fiber sensors in those cells is not completely the same.

Figure 5b shows a sensorgram of *δ*∆*f*_*rep*_ calculated by subtracting the green line (*δf*_*rep2*_) from the blue line (*δf*_*rep1*_) in Fig. 5a. The temperature drift almost disappeared, and only the step-like change with the sample RI was present in *δ*∆*f*_*rep*_. The mean and the standard deviation of *δ∆f*_*rep*_ were 0.76 ± 0.19 Hz at 0 vol% or 1.3165 RIU, − 7.58 ± 0.24 Hz at 1 vol% or 1.3179 RIU, − 16.48 ± 0.52 Hz at 2 vol% or 1.3193 RIU, − 25.64 ± 0.53 Hz at 3 vol% or 1.3207 RIU, − 34.59 ± 0.31 Hz at 4 vol% or 1.3222 RIU, and − 43.12 ± 0.34 Hz at 5 vol% or 1.3236 RIU, as shown by red plots in Fig. 5c. From these values, we calculated a relation between the sample RI and *δ*∆*f*_*rep*_. The linear relation between the sample RI and *δ*∆*f*_*rep*_ was obtained with a correlation coefficient (R) of 0.9999. The good fitting result indicated that the dual-comb effect minimizes the effect of temperature drift. From the slope coefficient of the linear fitting (= − 6216 Hz/RIU) and the stability of *δ∆f*_*rep*_ (= 0.198 Hz at 10 s, 0.627 Hz at 100 s, and 0.531 Hz at 1000 s based on three times the standard deviation), the RI resolution was determined to be 3.19 × 10^−5^ RIU at 10 s, 1.01 × 10^−4^ RIU at 100 s, and 8.54 × 10^−5^ RIU at 1000 s. In RI sensing of a liquid sample (glycerol solutions) different from another liquid reference (pure water), difference of thermo-optic effect between them may influence the effective RI and the temperature sensitivity because they have inherent thermo-optic coefficients (typically, 10^−4^ RIU/°C at 1550 nm)^40,41^. When their temperature is stable within a range of 0.1 °C, the drift of RI in the sample and the reference is estimated to be the order of 10^−5^ RIU. However, the temporal drift of thermo-optic coefficients will be common to each other if similar water solutions refer to the solutions used in the following measurements, and the active-dummy temperature compensation can work well. Therefore, the effect of different thermo-optic effects is negligible.

### Rapid detection of SARS-CoV-2 N protein antigen

Antibody modification of the intracavity MMI fiber sensor creates a photonic RF biosensor for the detection of target antigens through antibody–antigen reactions because the RI-dependent *f*_*rep*_ shift is converted into an antigen-concentration-dependent *f*_*rep*_ shift [see Fig. 1a]. We confirmed the effectiveness of the active-dummy compensation with the dual-comb configuration in high-precision RI sensing demonstrated above. The resulting enhanced RI precision covers the effective RI change expected by the antigen–antibody interaction on the sensor surface, enabling us to apply these dual sensing OFCs for rapid, high-sensitive detection of viruses/pathogens and biological molecules.

**Figure 6:**
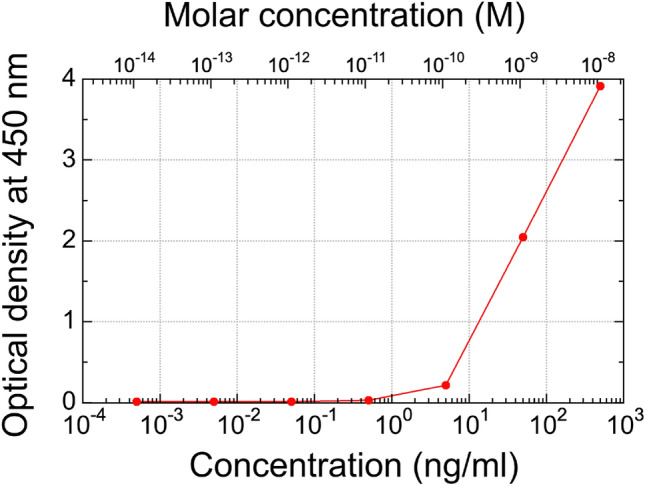
Affinity test between a commercialized antibody (FPZ0553, Fapon Biotech Inc.) and a commercialized antigen (FPZ0513, Fapon Biotech Inc.) for SARS-CoV-2 N protein by ELISA.

The concept of the antigen–antibody interaction (in this case, a viral protein) was applied for the detection of SARS-CoV-2 protein with dual-comb biosensing. Among several proteins in SARS-CoV-2, we selected the N protein instead of the well-known and peculiar spike protein for antigen–antibody interactions. This is because the N protein functions to package the viral RNA genome within the viral envelope into a ribonucleoprotein complex and has benefits such as abundance, low probability of mutation, and relatively low molecular weight. We used a pair of a commercialized N protein antibody (Fapon Biotech Inc., Dongguan, Guangdong, China, FPZ0553) and a commercialized recombinant N protein antigen (Fapon Biotech Inc., Dongguan, Guangdong, China, FPZ0513) for antigen–antibody interactions in the intracavity MMI biosensor [see Fig. 1b]. Before performing the dual-comb biosensing, we evaluate the affinity of this pair by enzyme-linked immunosorbent assay (ELISA). Figure 6 shows a relation between antigen concentration and optical density at 450 nm, indicating a sensitivity within a range of 10 pM to 10 nM in ELISA. We made a surface modification of the MMI fiber sensor (material = SiO_2_) with amino-terminated groups through a silane coupling reaction for a self-assembled monolayer (SAM) after surface cleaning and modifying by UV ozone. Then, the N protein antigen was immobilized on the amino-group-coated MMF fiber sensor surface to realize an active sensing OFC. Additionally, the SAM without immobilized antibody was applied to the surface of the MMI fiber sensor for the dummy sensing OFC. The MMI fiber sensors with and without surface modification by the immobilized antibody were placed together in the same sample cell for the active and dummy sensing OFCs, respectively [see the last row in Table 1 and Fig. 3d]. Solution samples of the N protein antigen in phosphate-buffered saline (PBS) at different molar concentrations were consecutively introduced into the sample cell with a peristaltic pump. The N protein antigen–antibody interaction could only occur on the sensor surface of the active sensing OFC because that of the dummy sensing OFC did not include immobilized antibodies. Thus, the dummy sensing OFC was used for the compensation of temperature drift assuming that non-specific adsorption did not occur on the sensor surface of the dummy sensing OFC.

**Figure 7:**
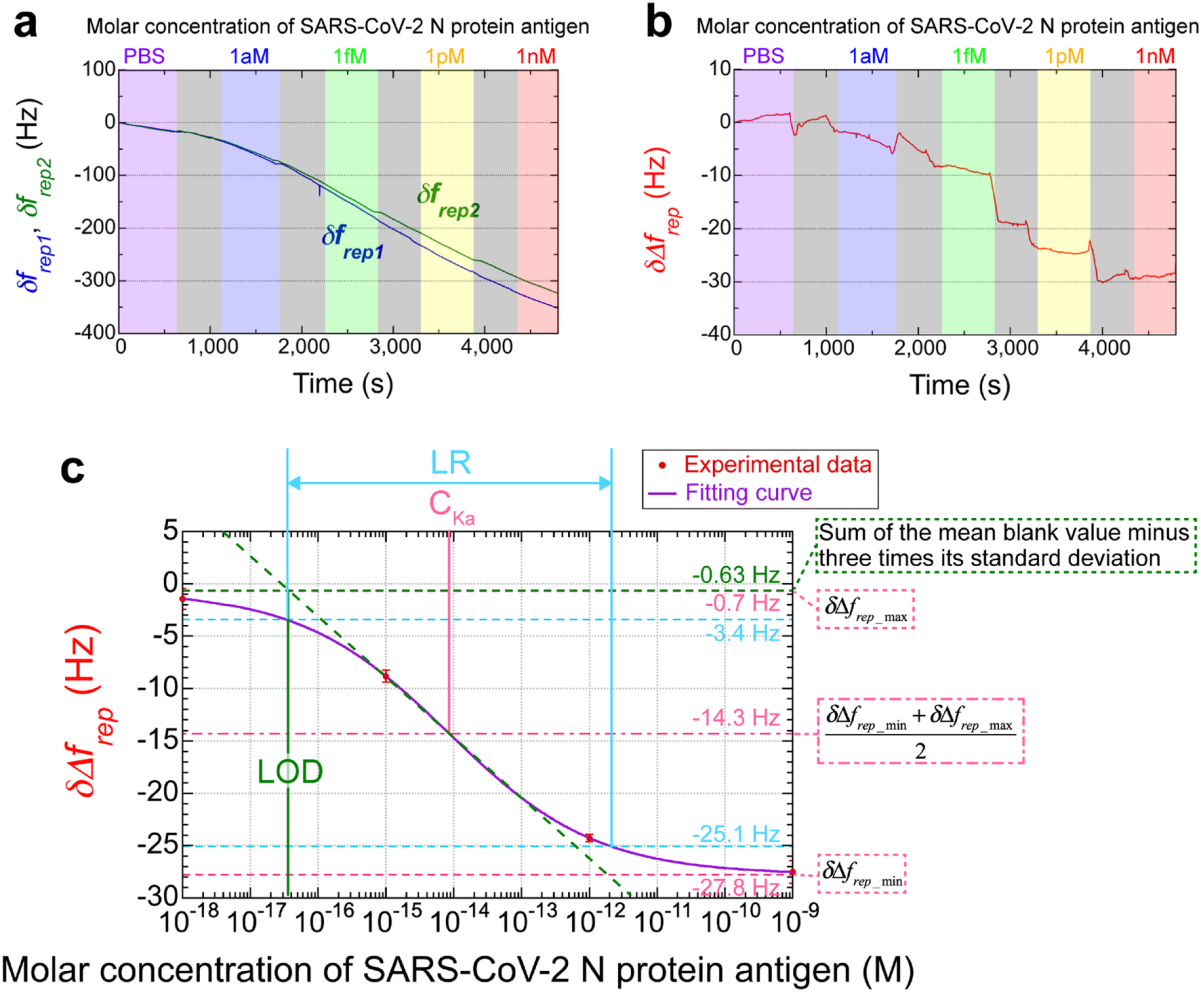
Rapid, high-sensitivity, dual-comb biosensing of the SARS-CoV-2 N protein antigen. (**a**) Sensorgram of *δf*_*rep1*_ and *δf*_*rep2*_ with respect to different molar concentrations of the SARS-CoV-2 N protein antigen. *δf*_*rep1*_ and *δf*_*rep2*_ were calculated as the frequency deviations from the initial values of *f*_*rep1*_ and *f*_*rep2*_, which were measured for the active biosensing OFC with immobilized antibody and the dummy OFC without immobilized antibody, respectively [see the last row in ﻿Table 1 and Fig. 3d]. Grey zones indicate the time period for sample exchange. (**b**) Sensorgram of *δ∆f*_*rep*_ with respect to different molar concentrations of the SARS-CoV-2 N protein antigen. *δ∆f*_*rep*_ was calculated as the frequency deviation from the initial value of *∆f*_*rep*_. (**c**) Relationship between the antigen molar concentration and *δ*∆*f*_*rep*_. Red circles show experimental data obtained from the sensorgram of *δ∆f*_*rep*_. The purple line shows the fitting curve for the sigmoidal function of the Hill plot.

Figure 7a shows the sensorgram of *δf*_*rep1*_ and *δf*_*rep2*_ as the molar concentration of the antigen/PBS solution increased from 1 aM (blue zone), 1 fM (green zone), 1 pM (yellow zone), and 1 nM (red zone) after starting with pure PBS (purple zone). We here selected the considerably wide range of molar concentrations for the initial evaluation of basic performance in dual-comb biosensing of SARS-CoV-2 N protein antigen. The precise evaluation focused within the clinically-relevant range of SARS-CoV-2 N protein antigen will be the future work. The time period for data acquisition [see color-highlighted zones in Fig. 7a] was set to 10 min. In the grey zones (time period = 8 min), we performed the following three steps: (1) we introduced the antigen/PBS solution into the sample cell with the peristaltic pump (1.5 min), (2) we waited for the antigen–antibody interaction to be completed (5 min), and (3) we rinsed the sensor surface with PBS to flush the accumulated N protein antigen that did not interact with the antibodies away (1.5 min). When the antigen was washed with the PBS buffer after being added and waited, the sensor signal reflects the amount of the antigen adsorbed on the sensor surface after desorption. However, the step-like change in *δf*_*rep1*_ with the antigen concentration was completely overshadowed by the background temperature drift. So, we next calculated the frequency difference (*δ∆f*_*rep*_) between *δf*_*rep1*_ and *δf*_*rep2*_ to eliminate the influence of temperature drift as described in the previous subsection. Figure 7b shows the sensorgram of *δ∆f*_*rep*_. Focusing on the zones highlighted in colors other than grey, a slightly dull stepped change in *δ∆f*_*rep*_ dependent on the molar concentration was observed, although a small drift in *δ∆f*_*rep*_ within the range of a few Hz remained at each molar concentration. To evaluate the validity of this behavior in the sensorgram, we calculated the mean and the standard deviation of *δ∆f*_*rep*_ at each molar concentration (each color zone other than grey zones): − 1.42 ± 1.00 Hz at 1 aM, − 8.82 ± 0.57 Hz at 1 fM, − 24.29 ± 0.36 Hz at 1 pM, and − 27.52 ± 1.08 Hz at 1 nM, respectively. They are indicated as red circles and corresponding error bars in Fig. 7c. The negative slope was consistent with the RI dependence of *δ∆f*_*rep*_ [see the red line plotted in Fig. 5c] because the progression of the antigen–antibody reaction increases the effective RI near the MMI fiber sensor and hence decreases *f*_*rep*_^42^. In this way, we demonstrated the potential for rapid detection of the SARS-CoV-2 N protein antigen within this range of molar concentrations.

## Discussion

To achieve the rapid, high-sensitivity detection of biomolecules, we developed dual-comb biosensing. Benefiting from the photonic-to-RF conversion in OFC, the enhanced sensitivity by the intracavity biosensor, and the active-dummy dual-comb compensation of temperature drift, the detection of the SARS-CoV-2 N protein antigen with a molar concentration from 1 aM to 1 nM was demonstrated in the measurement time of 10 min.

We first perform the quantitative analysis of the result shown in Fig. 7c. The antigen–antibody reaction is represented by a sigmoidal curve, often used when discussing the biosensor performance^43^; thus, the sigmoidal curve was applied to the experimental data to evaluate the ability of dual-comb biosensing to sense the SARS-CoV-2 N protein antigen. The sigmoidal function of Hill plot is given by

2 $$\delta \Delta f_{rep} = \delta \Delta f_{rep\_\min } + \frac{{\delta \Delta f_{rep\_\max } - \delta \Delta f_{rep\_\min } }}{{1 + \left( {\frac{\left[ C \right]}{{\left[ {C_{Ka} } \right]}}} \right)^{n} }},$$

where *δ∆f*_*rep_max*_ and *δ∆f*_*rep_min*_ are the maximum and minimum of *δ∆f*_*rep*_ within a range of 1 aM to 1 nM, *n* is the Hill coefficient, *C* is the concentration of the antigen, and *C*_*Ka*_ is the dissociation constant. The purple line in Fig. 7c represents the sigmoidal fit of the experimental data. Unfortunately, in this fitting analysis, as the number of unknown parameters of the sigmoidal curve (N = 4, *δ∆f*_*rep_min*_, *δ∆f*_*rep_max*_, *C*_*Ka*_, and *n*) is equal to the number of input data (N = 4, concentrations of 1 aM, 1 fM, 1 pM, and 1 nM), the degrees of freedom for the fitting are zero. In other words, results of this fit may be unreliable. The primary focus here is to roughly calculate performance parameters for biosensing using experimental data within specific concentration ranges. Based on the zero-degree-of-freedom fitting results, we will then proceed to engage in the following discussions. Addressing the issue of the zero-degree-of-freedom fitting results through detailed quantitative analysis will be part of our future work. From the curve fitting analysis of experimental plots with Eq. 2, we determined the following four parameters: *δ∆f*_*rep_min*_ =  − 27.8 Hz, *δ∆f*_*rep_max*_ =  − 0.7 Hz, *C*_*Ka*_ = 8.4 fM, and *n* = 0.40. *C*_*Ka*_ [see the pink solid line in Fig. 7c] is corresponding to the molar concentration at the middle [= − 14.3 Hz, see pink the dashdotted line in Fig. 7c] between *δ∆f*_*rep_max*_ and *δ∆f*_*rep_min*_ [see pink dashed lines in Fig. 7c]. When the linear range (LR) was defined as the molar concentration at 10 ~ 90% (= − 3.4 ~  − 25.1 Hz) of the dynamic signal range of *δ∆f*_*rep_max*_ to *δ∆f*_*rep_min*_ [see blue dashed lines in Fig. 7c]^43^, it was determined to be 34 aM ~ 2.1 pM [see blue solid lines in Fig. 7c]. Since the mean *δ∆f*_*rep*_ value (= 0.93 Hz) minus three times its standard deviation (= 0.52 Hz) of the blank sample (PBS) was − 0.63 Hz [see the purple zone in Fig. 7b], the LOD was calculated to be 37 aM from the crossover point [see the green solid line in Fig. 7c] between it and the linear approximation given by the Hill coefficient *n* (= 0.40).

We next compare the performance between the dual-comb biosensing and other biosensors when they are applied for biosensing of SARS-CoV-2 protein. While SPR benefits from the rapid analysis, its analytical sensitivity remains in LR of 2 ~ 1000 ng/mL with LOD of 1.02 pM^44^. The plasmonic enhancement by large gold nanoparticle decreases the LOD of SPR down to 85 fM or 4 pg/ml^14^. Also, the dual-functional plasmonic biosensor combining the plasmonic photothermal effect and localized SPR indicates LOD of 0.22 pM and LR of 0.1 pM ~ 1 μM^45^. In addition to these optical biosensors, colorimetric assay can be used for the detection of SARS-CoV-2 with a LOD of 0.18 ng/µL and a LR of 0.2 ~ 3 ng/µL within 10 min^46^. Dual-comb biosensing of SARS-CoV-2 N protein antigen achieves a LOD of 37 aM and a LR of 34 aM ~ 2.1 pM within 10 min in a simple matrix of PBS buffer that contains no interfering proteins, enzymes, and biomolecules. The above LOD and LR given in molar concentration correspond to concentrations of 1.7 fg/mL and 1.6 fg/mL ~ 99 pg/mL based on the molecular weight of the SARS-CoV-2 N protein antigen (= 47 kDa). When using complex matrices such as blood plasma or serum in place of PBS, testing time may increase depending on analyte diffusion and/or binding kinetics; still, it's within a range of 10 min. Table 2 summarizes the comparison of those biosensors for SARS-CoV-2 protein. In this way, the dual-comb biosensing greatly outperforms other biosensors in terms of LOD and LR.

**Table 2:** Comparison of testing methods for SARS-CoV-2.

Method	Limit of detection	Linear range	Time	Matrix	Target	References
SPR	1.02 pM	2 ~ 1000 ng/mL	–	PBS containing 0.1% Tween 20	N protein antigen	^44^
Nanoplasmonic-enhanced SPR	85 fM (4 pg/ml)	85 fM ~ 2 pM	5 min	PBS	N protein antigen	^14^
Dual-functional plasmonic biosensor	0.22 pM	0.1 pM ~ 1 μM	–	Nuclease-free water	Nucleic acid	^45^
Colorimetric assay	0.18 ng/µL	0.2 ~ 3 ng/µL	10 min	RNase H reaction buffer	N phosphoprotein gene	^46^
Dual-comb biosensing	37 aM (1.7 fg/ml)	34 aM ~ 2.1 pM (1.6 fg/ml ~ 99 pg/ml)	10 min	PBS	N protein antigen	–

Since we demonstrated the dual-comb biosensing of SARS-CoV-2 N protein antigen using highly purified, synthetic laboratory samples, the demonstrated results could not guarantee about the real specificity in the detection, which is another important performance of biosensing. For a real assessment of the biosensing performance including the specificity, a sample of complex matrices such as blood plasma or serum should be evaluated. In this case, non-specific adsorption of other proteins on the sensor surface spoils the specificity of dual-comb biosensing. We have to make special surface modifications designed to avoid non-specific adsorption in the active and the dummy sensing OFCs together with a specific antibody of SARS-CoV-2 N protein enhancing the specificity. Use of nucleic acids (DNA probe)^47^, in place of antibody–antigen, as biomolecular interaction also enables us to enhance the specificity of dual-comb biosensing largely. Work is in progress to enhance the specificity of the dual-comb biosensing by these approaches.

We finally discuss the potential for dual-comb biosensing to be used for the detection of other biomolecules of interest. The achieved *C*_*Ka*_ (= 8.4 fM) is considerably low, enabling its easy use for a wide variety of biosensing applications. For example, it has an option for early detection and quantification of cancer cells from a droplet of blood or other body fluids by detecting a sugar chain specifically expressed on the surface of cancer cells. Such liquid biopsy^48^ will be a powerful tool for detection of important biomarkers, such as proteins or RNA, in addition to cancer cells. Furthermore, if the molecular identification in biosensing is implemented by DNA probes in place of antigen–antibody reactions, it enables biosensing of exosomes via miRNA. As exosomes play an important role for intercellular communication, the biosensing of them is expected to make a great contribution to the diagnosis (marker) and treatment (drug delivery) of diseases such as cancer and Alzheimer's disease.

## Conclusion

We have demonstrated the dual-comb biosensing for rapid, high-sensitivity detection of biomolecules. To the best of our knowledge, this is the first application of an OFC as a biosensor itself. The integration of photonic-to-RF conversion, an intracavity sensor, and active-dummy dual-comb compensation in the OFC enables detection of the SARS-CoV-2 N protein antigen with an LOD of 37 aM in a measurement time of 10 min. The current COVID-19 pandemic may diminish in the near future; however, we are always at risk of facing another emerging and re-emerging infectious disease again. As dual-comb biosensing can be used for other viruses through selection of the antigen–antibody reaction or other molecular identifications, it will be important as a proactive measure against unknown infectious diseases. Simultaneous achievement of high sensitivity and rapid measurement by the dual-comb biosensing will greatly enhance the applicability of biosensors to viruses, biomarkers, environmental hormones, and so on.

## Materials and methods

### MMI fiber sensor

Figure 1b shows a schematic diagram of the intracavity MMI fiber sensor with antibody surface modification. When the surface of the fiber sensor is not modified with an antibody, the MMI fiber sensor functions as an RI sensor. The MMI fiber sensor is composed of a clad-less multimode fiber (MMF; Thorlabs Inc., Newton, NJ, USA, FG125LA, core diameter = 125 μm, fiber length = 58.94 mm) with a pair of single-mode fibers (SMFs) at both ends (Corning Inc., Corning, NY, USA, SMF28e + , core diameter = 8.2 μm, cladding diameter = 125 μm, fiber length = 150 mm)^34–36^. Only the exposed core of the clad-less MMF functions as a sensing part. The OFC light passing through the input SMF is diffracted at the entrance face of the clad-less MMF and then undergoes repeated total internal reflection at the boundary between the clad-less MMF core surface and the sample solution. Only the OFC modes satisfying the MMI wavelength *λ*_*MMI*_ can exit through the clad-less MMF and then be transmitted through the output SMF. *λ*_*MMI*_ is given by

3 $$\lambda_{MMI} = \frac{{n_{MMF} m_{MMI} }}{{L_{MMF} }}\left[ {D\left( {n_{sam} } \right)} \right]^{2} ,$$

where *L*_*MMF*_ and *n*_*MMF*_ are the geometrical length and RI of the clad-less MMF, *m*_*MMI*_ is the order of the MMI, *n*_*sam*_ is the RI near the clad-less MMF core surface (namely, sample RI), and *D(n*_*sam*_*)* is the effective core diameter of the clad-less MMF. Since *D(n*_*sam*_*)* is influenced by the Goos-Hänchen shift on the core surface of the clad-less MMF, *λ*_*MMI*_ is a function of the sample RI near the sensor surface. The intracavity MMI fiber sensor in this study functions as an RI-dependent optical bandpass filter tunable around *λ*_*MMI*_ (= 1556.6 nm) with constructive interference at *m* = 4. This *λ*_*MMI*_ was selected to match a spectral peak of the fiber OFC, suppressing the power loss. The RI-dependent *λ*_*MMI*_ shift of the OFC is converted into an RI-dependent *f*_*rep*_ shift via the wavelength dispersion of the cavity fiber [see Fig. 1a]. Furthermore, if the surface of the MMI fiber sensor is modified with a virus antibody, then the RI-dependent *f*_*rep*_ shift is converted into a virus-antigen-concentration-dependent *f*_*rep*_ shift through antibody–antigen reactions. In other words, the intracavity MMI fiber sensor with antibody surface modification enables a photonic RF biosensor for viruses.

### Single-comb configuration of the sensing OFC

We used a linear fiber cavity mode-locked by a saturable absorber mirror for easy, stable, mode-locked oscillation and compact size [see Fig. 2a]. The linear cavity includes a 2.6-m-long SMF (SMF; Corning Inc., Corning, NY, USA, SMF28e + , dispersion at 1550 nm = 17 ps km^−1^ nm^−1^), a 0.6-m-long erbium-doped fiber (EDF; nLIGHT Inc., Camas, WA, USA, LIEKKI ER30-4/125, dispersion at 1550 nm =  − 22.75 ps km^−1^ nm^−1^), a saturable absorber mirror (BATOP GmbH, Jena, Germany, SAM-1550–55-2 ps-1.3b-0, high reflection band = 1480–1640 nm, absorbance = 55%, modulation depth = 2.4%, relaxation time constant =  ~ 2 ps, size = 1.3 mm width, 1.3 mm height, 0.4 mm thickness), a wavelength-division-multiplexing coupler (WDM; AFR Ltd., Zhuhai, China, WDM-1–9855-N-B-1-F), a pumping laser diode (LD pump source; Thorlabs Inc., Newton, NJ, USA, BL976-PAG700, wavelength = 976 nm, power = 700 mW), a 90:10 fiber output coupler (OC; AFR Ltd., Zhuhai, China, PMOFM-55–2-B-Q-F-90), and an intracavity MMI fiber sensor (MMI). The total dispersion of the fiber cavity was set to − 0.12 pm/s^2^ for stable operation. The fiber cavity was placed in an aluminum box, and its temperature was not actively controlled. The light output of the sensing OFC was detected by a photodetector (PD; Thorlabs Inc., Newton, NJ, USA, PDA05CF2, wavelength = 800 ~ 1700 nm, frequency bandwidth = 150 MHz), and the resulting frequency signal of *f*_*rep*_ was measured by an RF frequency counter (Keysight Technologies, Santa Rosa, CA, USA, 53230A, frequency resolution = 12 digit s^−1^) synchronized to a rubidium frequency standard (Stanford Research Systems Inc., Sunnyvale, CA, USA, FS725, frequency = 10 MHz, accuracy = 5 × 10^−11^, and instability = 2 × 10^−11^ at 1 s).

### Dual-comb configuration of active and dummy sensing OFCs

We used a pair of linear-cavity sensing OFCs (frequency spacing = *f*_*rep1*_ and *f*_*rep2*_, frequency difference between them = *∆f*_*rep*_ = *f*_*rep1*_ − *f*_*rep2*_) for the active sensing OFC and the dummy sensing OFC in the dual-comb configuration [see Fig. 3a]. The configuration of each linear cavity was similar to that in Fig. 2a. The output light of the LD pump source was split into two beams and used for these two OFCs, which eliminates the influence of power drifts in the LD pump source through common-mode behavior. These OFC fiber cavities were enclosed in an aluminum box. Specification of the active and dummy sensing OFCs are shown in Table 1. The light output of the dual OFCs was detected by a pair of PDs, and the resulting frequency signals of *f*_*rep1*_, *f*_*rep2*_ and *∆f*_*rep*_ were measured through a combination of an RF frequency counter and a rubidium frequency standard.

### SARS-CoV-2 nucleocapsid protein antigen

The recombinant SARS-CoV-2 nucleocapsid protein, expressed in Escherichia coli, was used as an antigen. The molecular weight of 47 kDa was calculated based on the amino acid sequence of 419 residues using the molecular weight calculation tool provided by Expasy^49^. We further validated this by referring to literature that documented the utilization of a similar N capsid protein^50,51^. The slight variation in molecular weight compared to previous literature is thought to be attributed to differences in the His-Tag sequence and/or uncertainties in band reading on SDS-PAGE.

### Enzyme-linked immunosorbent assay (ELISA)

Recombinant SARS-CoV-2 nucleocapsid protein antigen (FPZ0513, 2 μg/ml) in phosphate-buffered saline (PBS) was added to 96-well EIA/RIA plates (Corning Inc., Corning, NY, U.S.) and incubated overnight at room temperature. Non-specific sites were blocked with Blocking One (Nacalai Tesque, Inc., Kyoto, Japan). After the plates were washed, diluted anti-SARS-CoV-2 nucleocapsid monoclonal antibody (FPZ0553) was added. After 1 h incubation at room temperature and washing the plates, goat anti-mouse IgG, (H + L) horseradish peroxidase conjugated (Invitrogen) was used as the secondary antibody. After washing the plates two times, TMB substrate solution (Invitrogen, Thermo Fisher Scientific Inc., Waltham, MA, U.S.) was added. The OD at 450 nm for each well was measured using a SpectraMax ABS microplate reader (Molecular Devices, San Jose, CA, U.S.).

### Antibody modification of the MMI fiber sensor

A schematic diagram of the intracavity MMI fiber sensor with antibody surface modification is shown in Fig. 1b. First, a UV ozone cleaner (Sun Energy Corp., Minoo, Osaka, Japan, SKB1101N-01) was applied to the MMI fiber sensor for 30 min to remove any organic compounds on the surface of the clad-less MMF and modify the resulting surface with hydroxy groups. Second, the surface of the clad-less MMF was modified with amino-terminated groups through a silane coupling reaction using 1% (v/v) 3-aminopropyltriethoxysilane (APTES) in ethanol for 1 h, followed by washing with ultrapure water and drying at 110 °C for 10 min. Third, for the antibody immobilization step by amide bonding, a dehydro-condensation reaction was used, which is a one-step reaction with higher reaction efficiency even in buffer solution, instead of the conventional method using carbodiimide activation^52^. The monoclonal antibody specific for the N protein antigen was immobilized on the amino-group-coated MMF core by a dehydration-condensation reaction using 10 mM 4-(4,6-dimethoxy-1,3,5-triazin-2-yl)-4-methylmorpholinium chloride (DMTMM) in PBS buffer (pH 7.4)^53^.

### Data analysis

The frequency spacings of the OFCs (*f*_*rep*_, *f*_*rep1*_, and *f*_*rep2*_) were continuously acquired by an RF frequency counter with a gate time of 100 ms and a sampling interval of 2.8 s. The frequency difference *∆f*_*rep*_ between *f*_*rep1*_ and *f*_*rep2*_ was calculated from acquired *f*_*rep1*_ and *f*_*rep2*_. Finally, *δf*_*rep*_, *δf*_*rep1*_, *δf*_*rep2*_, and *δ∆f*_*rep*_ were calculated as the frequency deviations from the initial values of *f*_*rep*_, *f*_*rep1*_, *f*_*rep2*_, and ∆*f*_*rep*_, respectively. In the dual-comb biosensing of SARS-CoV-2 N protein antigen, we calculated the 99.9% confidence interval for the first 100 data of the *δ∆f*_*rep*_ sensorgram measured in the PBS, and then used it as a criterion of rejection test to judge whether *δ∆f*_*rep*_ value acquired at each molar concentration is considered as a measurement error.

## Data Availability

Data underlying the results presented in this paper are not publicly available at this time but may be obtained from Takeshi Yasui upon reasonable request.
